# Preoperative Optimization for Elective Surgery in Crohn’s Disease: A Narrative Review

**DOI:** 10.3390/jcm14051576

**Published:** 2025-02-26

**Authors:** Karl Hazel, Rachel Cooney

**Affiliations:** 1 Department of Gastroenterology, Queen Elizabeth Hospital, University Hospitals Birmingham NHS Foundation Trust, Birmingham B15 2GW, UK; rachel.cooney@uhb.nhs.uk; 2 University of Birmingham, Birmingham B15 2TT, UK

**Keywords:** Crohn’s disease, inflammatory bowel disease, exclusive enteral nutrition, parenteral nutrition, prehabilitation, surgery, preoperative optimization

## Abstract

Crohn’s disease is a chronic inflammatory bowel disease and, despite an increase in the available drug treatments, many patients will still require surgery at some point in their disease course. Stricturing and penetrating phenotypes of Crohn’s disease are less likely to respond to our current medical treatment and, therefore, surgical intervention may be required. This is most commonly elective, planned surgery, thereby affording the opportunity to optimize medications, nutritional and inflammatory status, and steroid use. Poor nutritional status and previous surgery increase the risk of postoperative complications. Preoperative optimization has three main goals: reduction of postoperative complications; reduction of reoperation rates; and reduction of postoperative recurrence rates. A literature search was completed using PubMed, Embase, and Ovid using the search term “preoperative optimization in Crohn’s disease”, and it included both adult and pediatric studies, excluding those for perianal Crohn’s disease. In this narrative review, we examine the role of nutritional intervention, medical optimization pre and postoperatively, and the role of personalized prehabilitation in the reduction of postoperative complications. We demonstrate that these may all yield better postoperative outcomes for patients with Crohn’s disease undergoing elective surgery, although the evidence is somewhat limited and there is a requirement for more prospective randomized controlled trials to implement their role into standard practice or guidelines.

## 1. Introduction

Crohn’s disease (CD) is a chronic idiopathic inflammatory bowel condition. It is characterized by skip lesions and transmural inflammation and may affect any part of the gastrointestinal tract (GIT), from the mouth to the anus. It commonly presents with diarrhea, abdominal pain, weight loss, and fatigue [1]. Previous population-based cohort studies have shown that 30% of patients with CD have significant intestinal damage at diagnosis. Up to 50% of CD patients will require surgery within 20 years of diagnosis [2,3]. The etiology of CD remains unclear, but a combination of genetic, immunological, and environmental factors contributes to the onset of CD and its progression [4]. Gut dysbiosis is a common feature of CD and a Western diet appears to play a role in its development. This shift from high-fiber, low-fat foods to highly processed foods that are low in fiber, high in fat, and contain additives may have caused alterations to the host-gut microbiota relationship [5]. While genetics play a role in the distribution of GIT inflammation, the severity and phenotype of disease are influenced by other factors [6].

Stricturing CD may be present in up to 60% of patients and poses a particular therapeutic challenge [7]. Endoscopic balloon dilatation of short strictures, stricturoplasty, or surgical resection of the affected segment remain the only therapeutic options available for the treatment of fibrostenosing disease. These treatments are, however, associated with recurrence and requirement for repeated dilatation or successive resections. The most common surgical intervention in CD patients is ileocolectomy/ileocecectomy [8]. Recurrence of fibrostenotic disease following resection is common, with a wide range of reported frequencies as a consequence of differing durations of follow-up and varying definitions of recurrence, including endoscopic recurrence, symptomatic recurrence, or the need for further surgery [9,10,11]. Multiple resections for recurrent disease can lead to a number of sequelae, including malnutrition, short gut syndrome/intestinal failure, decreased quality of life, and increased inpatient stay, with associated costs to the patient and the healthcare system [8]. Indications for surgery are expanding beyond management of complications from bowel wall damage, i.e., strictures and fistulae.

The European Crohn’s and Colitis Organisation (ECCO) and European Society of Coloproctology (ESCP) consensus guidelines suggest that “surgery should be considered at an early stage in those with penetrating or fistulating disease and those with localized ileocaecal disease and obstructive symptoms but no significantly active inflammation” [12]. The Laparoscopic Ileocaecal Resection Versus Infliximab for Terminal Ileitis in Crohn’s Disease (LIR!C) study demonstrated that primary ileocaecal resection in patients with isolated ileocaecal disease had similar quality of life scores one year post-surgery when compared to those receiving medical treatment alone [13].

## 2. Methods

This is a narrative review of the current literature on the preoperative optimization of patients with Crohn’s disease. A search was completed using PubMed, Embase, and Ovid using the search term “Preoperative optimization of Crohn’s disease”. There was no time limitation on the search string. The definition of surgery in this review is limited to bowel surgery in Crohn’s disease and does not include perianal fistula surgery, and these studies were excluded from the search results. High-quality papers including systematic reviews and meta-analyses, randomized controlled trials, observational data, and the current European Crohn’s and Colitis Organisation (ECCO) guidelines were included. Conference papers were excluded. Pediatric studies were included as a significant body of evidence for the use of dietary interventions comes from pediatric populations. This narrative review focuses on the preoperative optimization of Crohn’s disease patients from a nutritional and pharmacotherapeutic perspective and the role of preoperative nutrition in preventing postoperative recurrence.

## 3. Timing of Surgery

Time is required to deliver good preoperative optimization; therefore, emergency surgery makes this impossible. The 2024 ECCO Guidelines on Therapeutics in Crohn’s Disease: Surgical Management recommend against emergency surgery in Crohn’s disease [14]. This is supported by a meta-analysis of cohort series that included 75,971 Crohn’s patients from 15 countries. This meta-analysis reported significantly lower mortality rates in patients who had elective [0.6%; 95% CI: 0.2–1.7%] when compared to emergent surgery [3.6%; 95% CI: 1.8–6.9%]. This highlights the importance of optimizing patients prior to surgery and avoiding, where possible, emergent surgery. The guidelines also recommend that once preoperative optimization has been initiated, it should then be followed by a reassessment of the patient to determine if they are suitable for, or continue to require, surgery. This approach is supported by a meta-analysis demonstrating that emergency bowel resection is associated with an increased risk of postoperative complications overall and abdominal septic complications. Another multicenter, observational study highlighted that there is an increased risk of postoperative complications and abscess recurrence in patients presenting with intra-abdominal abscesses undergoing emergency intervention. In addition, patients undergoing emergency surgery have a higher risk of stoma formation. Patients having emergency laparoscopic surgery have a higher conversion rate and are more likely to have longer segments of small bowel resected; a concern in CD due to the lifetime risk of short bowel syndrome [14]. Acute severe colitis and peritonitis are rare in Crohn’s disease, so avoidance of emergency surgery is possible in the majority of patients.

In 2024, the recommendations from the National Confidential Enquiry into Patient Outcome and Death (NCEPOD) “Making the Cut” study for patients undergoing Crohn’s disease study were published. They recommended that surgery should be recognized as a necessary treatment option rather than a failure of medical management and should be undertaken sooner in Crohn’s disease patients, and once this decision has been made, it should be undertaken within four weeks. A final recommendation advised that continuous involvement of the IBD team would promote joined-up care postoperatively [15].

## 4. Preoperative Nutritional Assessment in Crohn’s Disease

Patients with IBD frequently suffer from malnutrition and it is more commonly seen, or to a greater extent, in CD patients than UC [16]. The 2020 ECCO topical review examining perioperative dietary therapy in inflammatory bowel disease recommended that all patients should be screened for malnutrition and that a nutritional assessment should be performed if required, with a minimum of body mass index (BMI). It also recommended the correction of over- or undernutrition despite a paucity of evidence currently available [17]. Nutritional optimizations prevent malnutrition, micronutrient deficiencies, and osteoporosis, promoting optimal growth and development in pediatric patients [18]. The current European Society for Clinical Nutrition and Metabolism (ESPEN) guidelines also advise that all patients be screened for malnutrition at the time of diagnosis and then at regular intervals [19]. Malnourished patients with inflammatory bowel disease are more likely to suffer complications and require hospitalization due to infection; and, if hospitalized, they are more likely to have a prolonged admission and to require non-elective surgery and longer admissions, and to have an increased mortality and postoperative complication rates [20,21].

A significant proportion of IBD patients will lose lean muscle mass over time and sarcopenic obesity is becoming more commonly recognized. Corticosteroids contribute to net protein loss in adult patients with Crohn’s disease and, therefore, enteral nutrition may be beneficial in slowing or preventing protein turnover in patients receiving steroids [17]. The current ESPEN guidelines advise a daily intake of 1 g of protein per kilogram of body weight or 1.2 to 1.5 g/kg in patients with active inflammation in response to the proteolytic and catabolic state [19]. This is particularly true in patients who may have severe disease and require surgical intervention.

## 5. Role of Exclusive Enteral Nutrition and Other Diets in Preoperative Optimization

The following section will be broken into three subsections, examining the roles of exclusive enteral nutrition (EEN), partial enteral nutrition (PEN), and the Crohn’s disease elimination diet. Table 1 outlines key preoperative dietary intervention trials and their outcomes.

### 5.1. Exclusive Enteral Nutrition

Exclusive enteral nutrition is when patients replace their habitual diet with an exclusively liquid diet. EEN can take the form of elemental or semi-elemental formulations, with elemental forms having poor palatability [28]. Polymeric (whole protein) and semi-elemental (peptide) formulas are better tolerated. The most frequently used forms of EEN are polymeric feeds such as Fortisip^TM^, Ensure^TM^, or Modulen^TM^ [29]. While the clinical use of EEN is limited in the adult population, it has been a mainstay of treatment in pediatric CD as first-line therapy for induction of remission, thereby avoiding steroid use [30,31]. A Cochrane review showed that EEN was effective in an adult population but inferior to steroids, perhaps due to lack of compliance with EEN [32]. Evidence for EEN reducing intraoperative complications is limited to retrospective cohort studies and case-control studies [22,33,34].

Heerasing et al. demonstrated that those commenced on EEN when compared with matched controls had a shorter operating time [3.0 (2.5–3.5) vs. 3.5 (3.0–4.0) h respectively; *p* < 0.001] and were nine times less likely to develop an abscess or anastomotic leak [OR 9.1; 95% CI (1.2–71.2); *p* = 0.04]. This study also demonstrated that patients treated with preoperative EEN had a lower CRP than matched controls at the time of surgery [36 mg/L at baseline (IQR: 13–91) vs. 8 mg/L preoperatively (IQR 4–31); *p* < 0.001] and a quarter of patients avoided surgery [22]. Li demonstrated that patients who were immunosuppressant-free preoperatively and given EEN had a lower rate of stoma formation, reduced incidence of postoperative complications, and a reduced need for an urgent operation [34].

A small prospective study by Wang et al. included fifteen patients, with ten patients receiving preoperative EEN for a median duration of 41.5 days (15–70 days). During the course of EEN, there was a significant reduction in mean HBI (8.7 ± 1.9 versus 4.1 ± 2.4; *p* = 0.001) and CRP (11.7 ± 10.3 versus 0.8 ± 0.8 mg/dL; *p* = 0.008) and an increase in serum albumin (3.1 ± 0.6 versus 4 ± 0.6 g/dL; *p* = 0.022). Following EEN, two patients no longer required surgical intervention. When the EEN and immediate surgery groups were compared, the incidence of postoperative complications, hospital length of stay, clinical and endoscopic recurrence were similar. Patients receiving EEN, despite being malnourished at baseline, did not have an increased risk of postoperative complications when compared to patients who were well-nourished [23].

A systematic review in 2021 by Gordon-Dixon et al., regarding the use of preoperative EEN in Crohn’s disease, found that the current data on the use of EEN for preoperative optimization are of poor quality and that studies are underpowered to demonstrate significance [35]. Seven studies were included in the final analysis, including five retrospective cohort studies and two retrospective case-control studies. The review concluded that, in patients treated with EEN preoperatively, there were fewer infectious complications and a trend towards fewer stoma formations but only a single study included had significant power to demonstrate significance [35]. Use of preoperative EEN was not associated with improvements in body mass index or hemoglobin but there was a trend towards improvement in C-reactive protein and albumin levels. This reduction in infectious complications was further supported by a meta-analysis by Krasnovsky et al., published in 2024, showing that preoperative EEN is associated with reduced skin, soft tissue, and intra-abdominal infections. In all studies, there was no significant difference in Crohn’s recurrence rates at twelve months and the quality of the studies were either medium or poor [24].

EEN may have the potential to reduce the need for surgery. Hu et al. have shown that twelve weeks of EEN can effectively relieve inflammatory strictures [25]. In this prospective study, stricturing disease was suggested through patient-reported symptoms and confirmed radiologically and, where possible, endoscopically. A preliminary differentiation between fibrous and inflammatory strictures was determined using serum markers of inflammation, radiological “string sign”, and thickened bowel wall on CT. CT was performed at baseline and at week 12. A twelve-week course of EEN was administered as an outpatient treatment, with most patients receiving between 1500 to 2000 mL per day. A total of 50 out of the 65 eligible patients completed the entire EN treatment, while nine required surgical intervention for progressive bowel obstruction. Some 48 (73.8%) of patients achieved symptomatic remission, with 35 (53.8%) achieving radiological remission, and 42 (64.6%) achieved clinical remission. Overall, 50 patients (76.9%) achieved partial remission, while 30 patients (46.2%) achieved clinical remission and did not require surgery [25]. The aforementioned retrospective study by Heerasing et al. showed that preoperative EEN in patients with penetrating and stricturing CD resulted in 25% of patients avoiding surgery and in those who did proceed to surgery, there was a reduction in postoperative complications [22]. Avoidance of surgery may, however, only be short-term. Marafini et al. looked at EEN in patients with small bowel stricturing CD and new subacute obstructive episodes and found that patients not randomized to EEN had a higher rate of intestinal resection within the first three months of follow-up, rather than evenly distributed over twelve months [36].

### 5.2. Partial Enteral Nutrition

There is also interest in the use partial enteral nutrition, which is better tolerated by patients as it avoids the taste fatigue experienced by those on EEN. PEN can either be supplemental oral nutritional supplements (ONS), for example in malnourished patients, or replacement ONS whereby a proportion of daily nutritional intake is replaced by ONS. Supplemental ONS is recommended by ESPEN for all malnourished or at risk of malnutrition CD patients undergoing surgery, but the role of replacement PEN in patients who are not underweight or at risk of malnutrition in the preoperative setting is unclear [19].

In their 2022 study, Meade et al. showed that oral EN (≥600 kcal/day for ≥2 weeks duration) was reasonably well tolerated and associated with a reduction in 30-day postoperative complications. They did, however, recommend that further randomized controlled trials are required to confirm their findings. In a cohort of 300 patients, 204 underwent nutritional optimization with oral EN (*n* = 173) or PN (*n* = 31). On multivariate analysis, the cohort of nutritionally optimized patients had favorable outcomes: all complications [OR 0.29; 0.15–0.57, *p* < 0.001], surgical complications [OR 0.41; 95% CI 0.20–0.87, *p* = 0.02], non-surgical complications [OR 0.24; 95% CI 0.11–0.52, *p* < 0.001], and infective complications [OR 0.32; 95% CI 0.16–0.66, *p* = 0.001] [26].

### 5.3. The Crohn’s Disease Exclusion Diet

The Crohn’s disease exclusion diet is also being examined in the preoperative setting. Phase 1 of the CDED diet involves 50% of nutritional intake coming from ONS [37,38]. Wall et al. have published a New Zealand feasibility study looking at RCT of CDED vs. EEN vs. standard care for patients who are not malnourished. They have shown that this study is feasible, and the diets tolerated and their final results are awaited [39].

## 6. Role of Parenteral Nutrition

In cases where the use of enteral nutrition is not possible, the use of parenteral nutrition (PN) is recommended for five days or more and has previously been shown to reduce postoperative complication rates to 15%, compared to 24.4% in those who did not receive nutritional support, but statistical significance was not reached [40]. A historic study by Lashner et al. demonstrated that patients receiving PN required a reduced length of small bowel resection of 20 cm when compared to the non-PN group [41]. Additionally, Ayoub has shown that sixty days of PN, when compared with placebo, significantly reduces rates of non-infectious complications when controlled for disease severity and malnutrition preoperatively, in addition to preoperative weight loss >10% in six months being an independent risk factor and predictor of postoperative complications [42]. The ECCO consensus paper does not, however, recommend deferring emergency surgery and suggests that nutritional status should only be optimized in patients in which surgery can be delayed. A recent retrospective cohort study by Sun et al. examined the impact of PN vs. EEN on postoperative adverse outcomes in Crohn’s patients undergoing surgery for penetrating Crohn’s disease [27]. This study examined the outcomes for 223 patients receiving EEN and 90 patients receiving TPN. The study indicated that patients receiving TPN had a higher risk of adverse outcomes, such as abscess and phlegmon formation, enterocutaneous, and fistula formation, when compared to patients receiving EEN alone (OR 4.241, CI: 1.567–11.478) [27].

## 7. Steroids and Perioperative Complications

Steroids may be used to control symptoms prior to elective surgery but are a risk factor for intra-abdominal septic complications [OR 1.99, CI: 1.54–2.57] [12,43]. They have also been shown to increase the risk of superficial and deep surgical site infections. They also delay wound healing and increase the risk of pneumonia, myocardial infarction, adrenal insufficiency, and prolonged intubation [44,45,46]. Steroids also increase the risk of readmission within thirty days by 58%, the risk of reoperation by 21%, hospital stay over thirty days by 31%, and the risk of mortality by 32% [47]. Two single-center prospective studies showed that the use of EEN preoperatively allowed up to 62.5% of steroid-dependent patients to successfully wean off steroids in the preoperative period [33,48]. A single-center prospective study noted that perioperative EEN prolongs the immunosuppressant-free, including steroids, interval, reduces the risk for urgent surgery and reoperation, and reduces postoperative complications [34,40,49].

While systemic corticosteroids are indicated for attaining remission in IBD, they are not recommended for maintenance therapy [50]. A study by Stuck et al. pooled the data from 71 controlled trials and showed that there was no increased risk of infection in patients on less than 10 mg of prednisolone. For patients receiving 10 mg daily, the overall rate of infectious complications was 12.7% when compared to 8% in controls (2111 patients vs. 2087 controls; RR: 1.6; 95% CI: 1.3–1.9; *p* < 0.001) [51]. Two further studies by Huang et al. and Subramanian et al. showed that corticosteroid use was associated with a high risk of intra-abdominal septic complications (OR 1.99; 95% CI: 1.54–2.57), all postoperative complications (OR 1.41, 95% CI: 1.07–1.87), and an increased risk for postoperative infectious complications (OR 1.68, 95% CI: 1.24–2.28), respectively [52,53]. The 2018 ECCO-ESCP Consensus on Surgery for Crohn’s Disease advised that prednisolone at a daily dose of 20 mg or equivalent for six weeks or more is a risk factor for postoperative complications and should be weaned if possible [12].

## 8. Preoperative Pharmacotherapy

The PUCCINI (Prospective Cohort of Ulcerative Colitis and Crohn’s Disease Patients Undergoing Surgery to Identify Risk Factors for Post-Operative Infection) trial was the largest multicenter prospective surgical cohort study to examine the safety of anti-tumor necrosis factor (anti-TNF) agents in the perioperative setting in IBD [54]. This study was designed in response to the French GETAID and REMIND prospective cohort studies [55,56]. The GETAID study showed a correlation with anti-TNF use less than three months prior to surgery and increased postoperative morbidity and mortality as well as intra-abdominal septic complications. It did not, however, assess preoperative serum drug concentrations [55]. The aim of the PUCCINI study was to determine if preoperative anti-TNF exposure is an independent risk factor for infectious complications in patients with IBD undergoing abdominal surgery, with a secondary aim of examining the effects of serum drug concentration on postoperative infectious complications [54]. A total of 947 patients were enrolled in the trial, with 387 (40.9%) patients using systemic corticosteroids within two weeks of surgery. Some 235 patients (24.8%) were using thiopurines. 382 patients (40.3%) had used anti-TNF within twelve weeks, with 136 (14.4%) on vedolizumab and 21 (2.2%) on ustekinumab. A total of 208 patients had detectable anti-TNF serum levels at the time of surgery. Anti-TNF exposure within twelve weeks of surgery was not associated with an increased risk of any infection or surgical site infection. Additionally, thiopurine exposure was not associated with postoperative complications. Smoking preoperatively was found to be a significant risk factor for both surgical site infection or any infection within the cohort. It represents one of the most significant modifiable risk factors for postoperative complications [54]. A 2023 retrospective analysis by Schnitzler et al. examined postoperative complications in 447 patients undergoing surgery for IBD between 2012 and 2022. Some 275 (61.3%) patients were exposed to biologics within twelve weeks of surgery, with 68.7% of patients receiving a biologic dose within four weeks of surgery. The study found that biologic exposure in the preoperative setting did not increase the risk of postoperative infectious complications [57].

The 2024 ECCO Guidelines on Therapeutics in Crohn’s Disease: Surgical Management, as mentioned previously, “recommend against cessation of biologics prior to surgery as the current evidence suggests that preoperative treatment with anti-TNF therapy, vedolizumab and ustekinumab does not increase the risk of postoperative complications in CD patients undergoing abdominal surgery” [14]. Data on the Janus kinase inhibitor and sphingosine 1 phosphate receptor modulators are lacking.

Communication between the medical and surgical team as well as involvement of the patient is key to ensure appropriate management of patients in the pre and postoperative periods. This is highlighted in the 2024 NCEPOD ‘Making the cut’ paper. The involvement of the wider multidisciplinary team, including pharmacists and IBD nurses, to ensure there is clarity regarding the timing of surgery and postoperative plans regarding medication, is essential for safe patient care [15]. They emphasized that all patients with Crohn’s disease should have access to holistic care and require careful medication management pre, intra, and postoperatively.

## 9. Surgical Prehabilitation

The American College of Surgeons (ACS) defines surgical prehabilitation as “a process of improving the functional capability of a patient prior to a surgical procedure so the patient can withstand any postoperative inactivity and associated decline” [58]. Currently, the evidence for prehabilitation in Crohn’s disease is lacking, with most studies focusing on nutritional intervention and optimization in the perioperative period. The term “prehabilitation” encompasses a wide range of interventions that aim to improve the preoperative status of patients, from a medical, physical, nutritional, and psychological perspective [59]. A systematic review by Jain et al., found that a trimodal prehabilitation program, including exercise and inspiratory muscle training, and nutritional and psychological support, improves a patient’s preoperative functional capacity and reduces postoperative complications following abdominal surgery [60]. The majority of patients included in this study were undergoing cancer surgery and it may not be directly applicable to the IBD cohort but it did emphasize the importance of exercise for building muscle, improving cardiopulmonary fitness, and reducing the debilitating effects of reduced mobility in the perioperative period [61].

A 2022 study by Ferrandis et al. showed that personalized prehabilitation reduces anastomotic complications when compared to upfront surgery prior to ileocolic resection in high-risk patients with Crohn’s disease [62]. This was a single-center retrospective study involving 90 patients that examined all high-risk patients undergoing ileocaecal resection with primary anastomosis over a ten-year period. Patients were classified as high-risk if they had one of the following risk factors; hypoalbuminemia < 30 g/L or weight loss > 10% of total body weight in the preceding six months, treatment with corticosteroids within four weeks of surgery, or the presence of preoperative intra-abdominal sepsis. Personalized prehabilitation included nutritional support, corticosteroid adjustment, and management of intra-abdominal sepsis for at least seven days preoperatively. Some 71% of patients received personalized prehabilitation for a median duration of 37 days, with the number of preoperative risk factors improved (1.21 vs. 1.06; *p* = 0.001). The 90-day anastomotic complication and reoperation rates were lower in patients that received prehabilitation; 6.25 vs. 23.1%; *p* = 0.031 and 3.1 vs. 19.2%; *p* = 0.019, respectively [62].

Overall, the evidence for prehabilitation in Crohn’s disease surgery is lacking and prospective studies are required to support its role as used in other surgical interventions in populations such as cancer or elderly patients.

## 10. Conclusions

We suggest that the mainstay of preoperative optimization for patients undergoing elective surgery for Crohn’s disease is centered around optimization of nutrition as there is the most significant body of evidence to support this, although larger prospective randomized controlled trials are required. While EEN in a pediatric setting has been shown to reduce the requirement for steroids, it remains difficult to implement in an adult population but may reduce the requirement for surgery [25,36]. Overall, the most significant benefit seems to be regarding reduced intraoperative and postoperative complications, namely infectious complications; although these data come from small, retrospective studies only [22,30,31,33,34]. The 2021 systematic review by Gordon-Dixon drew conclusions that preoperative use of EEN reduced infectious complications and potentially leads to fewer stoma formations, but studies are significantly underpowered in this setting to draw conclusions [35]. The 2022 Meade et al. study examining the use of oral EN or PN also demonstrated a reduction in the rates of surgical or 30-day postoperative infectious complications, but advised that additional studies would be required to confirm this [26]. While the use of PN may reduce the resection length, it may result in higher rates of adverse outcomes such as phelgmon and fistula formation when compared to EEN; as demonstrated by Sun et al. [27]. On this basis, EEN or PEN would be a preferable choice to nutritionally optimize patients preoperatively, particularly in the elective setting in which patients can receive EEN or PEN in the community and do not require hospital admission prior to surgery. This approach to preoptimization and elective surgery for CD rather than emergency surgery is supported by the 2024 ECCO Guidelines on Therapeutics in Crohn’s Disease: Surgical Management, which recommend against emergency surgery in Crohn’s disease [14].

The use of steroids preoperatively has also been shown to increase the risk of postoperative septic complications, delay wound healing, and increase readmission rates [12,43,47]. The 2018 ECCO-ESCP Consensus on Surgery for Crohn’s Disease advised that prednisolone should be weaned to a daily dose less than 20 mg, or stopped, at least six weeks preoperatively where possible [12]. The PUCCINI trial showed no increased postoperative complication or infectious risk associated with anti-TNF agents in the preoperative setting [54]. The 2024 ECCO Guidelines on Therapeutics in Crohn’s Disease: Surgical Management advise against the cessation of anti-TNF agents, vedolizumab, and Ustekinumab, and that, as such, they should be continued until surgery [14]. There is no clear guidance with regards to JAK inhibitors or S1P receptor modulators. The evidence for surgical prehabilitation in CD is lacking and requires further investigation with prospective RCTs, but it may show benefits in reducing postoperative complications as per the 2022 Ferrandis et al. study [62].

Preoperative optimization of patients undergoing surgery for Crohn’s disease is an example of collaborative multidisciplinary care. We conclude that corticosteroid doses should be reduced and stopped where possible, nutritional screening and optimization are key, and how best to deliver this is an area of ongoing research. Emergent surgery should be avoided where possible. Biologic medications should not be stopped in the perioperative period, as backed by international guidelines. Further research regarding newer advanced oral therapies is needed. Close communication between the IBD MDT with a patient-centered approach is key for successful surgical outcomes.

## Figures and Tables

**Table 1:** Main studies comparing postoperative outcomes of dietary interventions preoperatively in Crohn’s disease.

Authors [Ref]	Year	Design and Setting	Study Population	Number of Patients	Objective	Main Results
Heerasing et al. [22]	2017	Retrospective case-controlled study	Adult Crohn’s disease patients requiring urgent surgery for structuring or penetrating complications	51 patients treated with preoperative EEN	To test the hypothesis that exclusive enteral nutrition provides a safe and effective bridge to surgery and reduces postoperative complications	EEN frequently downstages the requirement for surgery EEN is associated with a reduction in systemic inflammation, operative times and the incidence of postoperative abscess or anastomotic leak
Wang et al. [23]	2016	Retrospective analysis of a prospectively-maintained database	Adult Crohn’s disease patients undergoing bowel resection for ileal or ileocolonic CD	81 patients; 42 EN, 39 normal diet	To determine the effect of preoperative exclusive EN on postoperative complications and recurrence after bowel resection in patients with active CD	Patients receiving EEN had improved BMI, albumin, CRP, and CDAI status compared to baseline after EEN therapy for 4 weeks Lower incidence of both infectious and non-infectious complications in patients receiving EEN compared with standard diet EEN therapy for 4 weeks reduced endoscopic recurrence rates 6 months postoperatively EEN did not reduce clinical recurrence 2 years postoperatively
Krasnovsky et al. [24]	2024	Systematic review and meta-analysis	Adult Crohn’s disease patients undergoing intestinal surgery for CD	1918; 874 EEN patients, 1044 controls	To quantify the association of preoperative EEN and TPN with surgical outcomes in patients undergoing intestinal surgery for CD	The relative risk of intra-abdominal septic complications decreased 2.1-fold in patients receiving preoperative EEN The risk of skin and soft tissue infection decreased 1.6-fold
Hu et al. [25]	2014	Single-center prospective observational study	CD patients with inflammatory strictures	65	To assess the effect of EEN therapy on relieving inflammatory strictures in CD	EEN therapy can effectively relieve inflammatory bowel strictures in CD EEN therapy improves inflammatory, nutritional, and radiologic parameters in CD patients with inflammatory strictures
Meade et al. [26]	2021	Single-center retrospective cohort study	CD patients undergoing ileal or ileocolonic resection	300 patients; 204 with nutritional optimization, 96 without	Preoperative EN in patients is associated with improved postoperative outcome	Oral EN for at least 2 weeks was reasonably well tolerated and associated with a reduction in 30-day postoperative complications
Sun et al. [27]	2024	Single-center retrospective cohort study	CD patients undergoing surgery for penetrating disease	510 patients; 101 TPN, 409 EEN	To assess the impact of preoperative EEN vs. TPN on the incidence of postoperative adverse outcomes	TPN increased the occurrence of postoperative adverse outcomes when compared to EEN EEN exhibited superior nutritional and surgical outcomes in comparison with those who received TPN
